# An Examination of the Long-Term Neurodevelopmental Impact of Prenatal Zika Virus Infection in a Rat Model Using a High Resolution, Longitudinal MRI Approach

**DOI:** 10.3390/v13061123

**Published:** 2021-06-11

**Authors:** Rita T. Patel, Brennan M. Gallamoza, Praveen Kulkarni, Morgan L. Sherer, Nicole A. Haas, Elise Lemanski, Ibrahim Malik, Khan Hekmatyar, Mark S. Parcells, Jaclyn M. Schwarz

**Affiliations:** 1 Department of Psychological and Brain Sciences, University of Delaware, Newark, DE 19716, USA; brennang@udel.edu (B.M.G.); nhaas@udel.edu (N.A.H.); lemanski@udel.edu (E.L.); jschwarz@udel.edu (J.M.S.); 2 Center for Translational Neuroimaging, Department of Psychology, Northeastern University, Boston, MA 02115, USA; p.kulkarni@northeastern.edu; 3 W. Harry Feinstone Department of Molecular Microbiology and Immunology, Johns Hopkins Bloomberg School of Public Health, Baltimore, MD 21205, USA; msherer2@jhmi.edu; 4 Center for Biomedical and Brain Imaging, Department of Psychological and Brain Sciences, University of Delaware, Newark, DE 19716, USA; imalik@udel.edu (I.M.); hekmat@udel.edu (K.H.); 5 Department of Animal and Food Sciences, University of Delaware, Newark, DE 19716, USA; parcells@udel.edu

**Keywords:** Zika virus (ZIKV), neuroimaging, MRI, neurodevelopment, pregnancy, congenital infection

## Abstract

Since Zika virus (ZIKV) first emerged as a public health concern in 2015, our ability to identify and track the long-term neurological sequelae of prenatal Zika virus (ZIKV) infection in humans has been limited. Our lab has developed a rat model of maternal ZIKV infection with associated vertical transmission to the fetus that results in significant brain malformations in the neonatal offspring. Here, we use this model in conjunction with longitudinal magnetic resonance imaging (MRI) to expand our understanding of the long-term neurological consequences of prenatal ZIKV infection in order to identify characteristic neurodevelopmental changes and track them across time. We exploited both manual and automated atlas-based segmentation of MR images in order to identify long-term structural changes within the developing rat brain following inoculation. The paradigm involved scanning three cohorts of male and female rats that were prenatally inoculated with 10^7^ PFU ZIKV, 10^7^ UV-inactivated ZIKV (iZIKV), or diluent medium (mock), at 4 different postnatal day (P) age points: P2, P16, P24, and P60. Analysis of tracked brain structures revealed significantly altered development in both the ZIKV and iZIKV rats. Moreover, we demonstrate that prenatal ZIKV infection alters the growth of brain regions throughout the neonatal and juvenile ages. Our findings also suggest that maternal immune activation caused by inactive viral proteins may play a role in altered brain growth throughout development. For the very first time, we introduce manual and automated atlas-based segmentation of neonatal and juvenile rat brains longitudinally. Experimental results demonstrate the effectiveness of our novel approach for detecting significant changes in neurodevelopment in models of early-life infections.

## 1. Introduction

Zika virus (ZIKV) is an arthropod-borne Flavivirus, closely related to dengue, yellow fever, Japanese encephalitis, and West Nile viruses. ZIKV is primarily transmitted to humans through the urban transmission cycle by mosquitos of the species *Aedes aegypti* which thrive in the densely populated tropical and subtropical regions of Asia, Africa, and the Americas [1,2,3]. More recently, ZIKV has also been found to be transmitted through other routes, including sexual transmission, transfusion of blood products, breast milk feeding, and vertical transmission from the pregnant mother to the fetus [4,5]. Since its emergence in the Americas in 2015, there has been renewed interest in understanding the pathogenicity of ZIKV in order to develop vaccines and therapeutic strategies able to combat infection. Nevertheless, no effective therapies currently exist.

ZIKV emerged in 2015 as a major challenge for global health agencies due to its ability to cause congenital Zika syndrome (CZS), which is characterized by brain abnormalities and microcephaly in neonates and cognitive developmental deficits in affected young children [6,7]. In fact, there has been nearly a 20-fold increase in the incidence of microcephaly and birth defects seen in infants delivered by women during the ZIKV outbreak in Brazil between 2014 and 2015 [8,9]. This prompted the Centers for Disease Control and Prevention (CDC) to officially declare a causal link between prenatal ZIKV infection and the serious brain abnormalities seen in affected infants. Although this link seems unquestionable, present findings in humans offer a limited scope of ZIKV’s full pathogenicity. Moreover, it is becoming increasingly apparent that solely investigating the consequences of Zika in children who show overt symptoms or physical deformities at the time of birth provides a very limited snapshot of the potential impact of this virus infection on cognitive function. 

Importantly, it has only been six years since the 2015 outbreak in Brazil, and so our ability to study the long-term consequences of Zika exposure in humans is limited, and therein lies a dilemma. Thus, it is necessary to utilize animal models that best mirror the symptoms, transmission, and outcomes associated with this virus in order to understand the long-term neurological consequences of prenatal ZIKV infection in all affected offspring. This approach will allow clinicians to identify very early on which individuals may be at risk for developmental delays and neurocognitive deficits later in life, even in infants that were asymptomatic at birth, so that these individuals can be recommended for occupational, physical, and cognitive therapeutic interventions that could hopefully rescue or mitigate the long-term consequences of the prenatal ZIKV infection.

While human and animal model studies demonstrate abnormalities in certain brain structures associated with ZIKV infection, it is not fully understood: (1) how structural changes in the brain correlate with active prenatal ZIKV infection and its associated replication, (2) when various neurological changes may begin to emerge throughout development, and (3) whether these neurological changes persist throughout the lifespan. In the present study, we utilize a longitudinal in vivo MRI technique and employ a dual approach of automated segmentation and manual segmentation for subsequent analyses to explore the long-term neurological sequelae of prenatal ZIKV infection in rat offspring.

## 2. Materials and Methods

### 2.1. Animals and Breeding

All experiments used Sprague Dawley rats; adult males were ordered from Envigo Laboratories (Indianapolis, IN, USA) and adult females were ordered from Charles River Laboratories (Wilmington, MA, USA). Animals were housed in same-sex pairs in clear, polypropylene cages (45 cm × 20.5 cm × 24 cm) with *ad libitum* access to food and water. The colony room maintained controlled temperature and humidity, at 22 °C under a 12:12 h light/dark cycle. Thirty nulliparous females aged 54–56 days were paired individually with male rats for breeding to generate litters throughout the course of the experiment. The presence of a vaginal plug was checked daily in order to confirm pregnancy and the date of conception which was considered embryonic day 0 (E0). Once pregnancy was confirmed, male breeders were removed from the breeding cage, and females were moved to a Biosafety Level 2 (BSL 2) animal isolation facility where they were individually housed in clean cages prior to infection on E18. Day of birth (DOB, approximately E23) was assigned as postnatal day 0 (P0). All experiments were approved by the University of Delaware Institutional Animal Care and Use Committee (Animal Use Protocol #1306) under the *Guide for the Care and Use of Laboratory Animals* of the National Institute of Health.

### 2.2. ZIKV Growth Conditions

Zika virus stocks (strain PRVABC059, Puerto Rico, Human, December 2015 from ATCC) were propagated in Vero cells and serially passaged to yield high-titer virus stocks. Stocks prepared for infection studies were prepared as T75 flasks of Vero cells infected at an MOI of ~1 with cells being incubated for 96 h prior to harvest (based on observed cytopathic effects). At 96 h postinfection, flasks were sealed with parafilm and snap frozen at −80 °C. Cells underwent three rounds of snap freezing–rapid thawing at 37 °C in a water bath. Cell supernatant medium and lysates were clarified by centrifugation at 1500 rpm for 10 min, and the supernatant was filtered through a 0.45 µM filter to remove cellular debris. This supernatant medium was the source of infectious virus, and viral titer was determined by serial dilution on Vero cells (10^2^–10^9^) in triplicate wells of 12-well dishes plated with Vero cells. Viral titer was counted by fixing of cell monolayers at 96 h postinfection with 1% paraformaldehyde in 1X PBS for 1 h, followed by three washes with 1X PBS and IFA using rat anti-Zika antibody (Kerafast Inc., Boston, MA, USA, cat. EDW003, rat #15, diluted 1:2000).

### 2.3. ZIKV Inactivation

Ultraviolet (UV) irradiation, which inactivates viruses by chemically modifying their genome, has been used successfully to inactivate many viruses, including ZIKV. In order to inactivate ZIKV, a portion (5 mL) of the total infectious supernatant medium was removed and placed in a 60 mm dish for UV inactivation. UV inactivation (254 nm) was performed using a Stratlinker crosslinker by placing the 60 mm dish (lid removed) inside the crosslinker set to 20,000 J, constant power for 10 min at room temperature. Following inactivation, the supernatant medium was collected and divided into 1 mL aliquots, and a portion was titrated on Vero cells, as above at 10^−1^ to 10^−4^ dilutions, in triplicate. As with infectious virus, monolayers were fixed at 96 h postinfection and stained by indirect immunofluorescence assay (IFA), as described above. Loss of infectivity was confirmed as only individual cells were IFA-positive due to inoculum uptake in the absence of replication and spread to other cells.

### 2.4. Inoculations

Pregnant females were inoculated at embryonic day 18 (E18) by subcutaneous injection into the loose skin over the interscapular area with a mock diluent control (0.1 mL of the same culture media used to grow ZIKV), ZIKV (dose of 1 × 10^7^ PFU in 0.1 mL culture media), or inactivated ZIKV (iZIKV) (inactivated dose of 1 × 10^7^ PFU in 0.1 mL culture media). The viral dose was selected based on previously published studies in mouse models and previously published results from our lab using a rat model [10,11,12,13]. iZIKV control was used to distinguish whether outcomes of ZIKV relied on active viral replication as opposed to the maternal immune response triggered by the presence of viral particles alone.

Infection on E18 of gestation was selected for the current study based on previous work in our lab indicating that during late gestation, the immune system of the pregnant female is significantly suppressed and therefore unable to respond effectively to an immune challenge, resulting in vertical transmission to the fetuses [10,14]. This model results in a significant febrile response in pregnant dams, a small but significant increase in pup death at the time of birth, and associated increases in cell death in the pups at the time of birth. Rats were inoculated on E18 based on the presence of a sperm plug on E1.

### 2.5. Pup Identification

Pups were identified by toe tattooing the plantar surface of the paws on P2. Toe tattooing is a reliable and permanent identification method that is less invasive than other alternatives. On postnatal day 2 (P2), pups were tattooed, along with the dam to prevent excessive maternal grooming of the tattoo in the pups. After being tattooed, the toes were gently wiped with dry gauze to remove any excess ink, and the animal was returned to its home cage, where it remained undisturbed. Rats were later weaned from the dam on P21 and separated into same-sex pairs in clean cages.

### 2.6. Image Acquisition

During the scanning protocol, rats were anesthetized in order for proper image acquisition. Before beginning any anesthesia procedures, all oxygen tanks and isoflurane levels were checked to ensure a sufficient amount for the day’s experiments. Rats were placed in an induction chamber with isoflurane set to either 2.5 or 3.0 L/min, based on the age of the animal. Once anesthetized, rats were removed from the chamber and placed in the scanning bed, secured with a nose cone. A bite bar was also used to secure juvenile and adult rats (P16–P60). After induction, isoflurane was then administered through the nose cone at 2.0 L/min for maintaining unconsciousness. Rats remained under anesthesia for no longer than 30 min for the completion of scanning. Once removed from the scanner, rats were placed in a recovery cage with a warm pad and constantly monitored until they fully recuperated from the effects of anesthesia. During the recovery phase, oxygen was administered at 0.5 L/min.

Images were obtained using a 9.4 T Bruker Biospec 94/20 small animal MR system (Bruker BioSpec MRI, Ettlingen, Germany). Each animal was imaged on four different occasions: P2, P16, P24, and P60. As individual organs, tissues, and structures mature at different paces, choosing the age and observation period for the animal model of interest must be done carefully. Therefore, in order to obtain a robust picture of both normative and aberrant neurodevelopment, we selected timepoints that were representative of four main stages of rodent development. Postnatal day 2 was chosen to signify the early neonatal stage, representing 0–28 days in humans [15,16,17]. Postnatal day 16 was chosen because it is representative of the late neonatal phase or infantile phase, equivalent to 1–2-year-old humans [15,16,17]. P24 was selected for analysis as it represents the prepubertal developmental timepoint, in which rats are considered juveniles and equivalent to 2–12-year-old humans [15,16,17]. P60 was selected for analysis as it represents adulthood, in which rats are considered to be fully developed and equivalent to 18-year-old humans [15,16,17]. Both mouse and rat brain 2 × 2 surface array coils were used; the mouse coil was used for the P2 and P16 age points, while the rat coil was used for the P24 and P60 age points. The correct positioning of each animal within the RF coil was confirmed through a series of localizer images. T2-weighted imaging was chosen for its sensitivity to a wide range of brain pathologies [18] and optimal display of anatomical features. During each imaging session, a high-resolution anatomical data set was collected using the rapid acquisition with relaxation enhancement (RARE) pulse sequence of 50 slices, 0.5 mm slice thickness, 30 mm field of view (FOV), 256 × 256, 5 s repetition time (TR), 32 ms echo time (TE), RARE factor of 8, and 5 min 20 s acquisition time.

### 2.7. Automatic Segmentation of ROIs

A magnetic resonance histology (MRH) atlas of the developing rat brain, courtesy of Duke Center for In Vivo Microscopy, was superimposed onto age-matched T2-weighted MR images in order to define the boundaries of 26 individual regions of interest (ROIs). The Ekam Visualization and Analysis (EVA) tool (Ekam Solutions LLC, Boston, MA, USA) accurately achieved alignment of both the MRH atlas and image through ITK registration framework with affine transform and mutual information based similarity matrix. Once the image was properly registered to the atlas, we segmented boundaries for each ROI. Each ROI was extracted using inverse transform matrix (T^−1^) in the original space of the MRI scan. Each MRI voxel was tagged into the specific brain region based on the brain atlas registration. 

In quantitative measurements, such as volume estimations of brain structures, it is important to consider the partial volume effect (PVE) which can arise when there is a mismatch in the atlas resolution and subject resolution. The resolution of the MRH atlas used for segmentation (0.059 × 0.059 × 0.25 mm) is much higher than the resolution of our MRI data (0.117 × 0.117 × 0.5 mm). This may introduce segmentation inaccuracies because multiple atlas voxels might occupy a single subject voxel. To accurately classify these voxels with partial volume effects, the ROI with the greatest occupied volume within the subject voxel was assigned to that specific voxel. The total volume of each region was calculated by multiplying the unit volume of the voxel in mm^3^ by the number of voxels. To account for different brain sizes, all the ROI volumes were normalized by dividing each ROI volume by the total brain volume for each rat.

### 2.8. Manual Segmentation of ROIs

**Figure 1:**
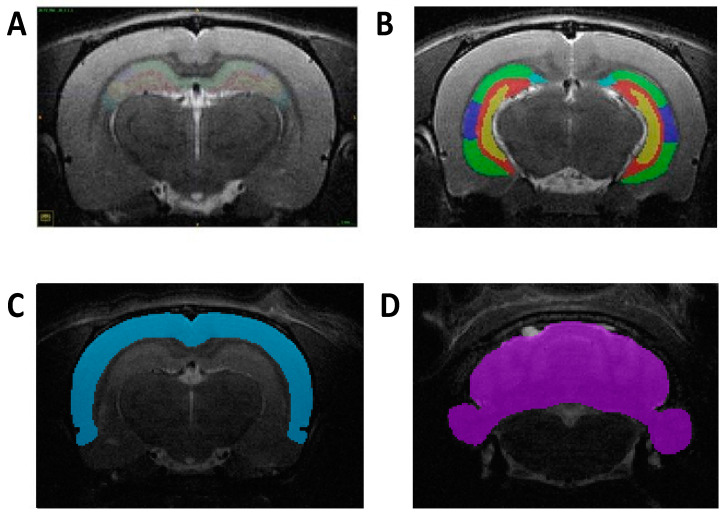
Representative images of manual segmentation in the coronal plane at P60. (**A**) Delineation of hippocampus and its subregions segmented at a more rostral level. (**B**) Delineation of hippocampus and its subregions segmented at a more caudal level. (**C**) Depiction of the isocortex region manually segmented. (**D**) Depiction of the cerebellum region manually segmented.

The whole hippocampus, CA1, CA2, CA3, dentate gyrus, isocortex, and cerebellum were traced using the Insight Toolkit SNAP program (v.3.8.0; www.itksnap.org) [19]. ROIs were identified using four atlases, each appropriately age-matched to the animal at the time of each of the four scans. *Atlas of the Developing Rat Nervous System* by George Paxinos, Ken W. S. Ashwell, and Istvan Tork (1994) served as reference for the P2 animal scans. *Atlas of the Developing Rat Brain in Stereotaxic Coordinates P14* by Roustem Khazipov et al. was used for P16 scans. *Atlas of the Developing Rat Brain in Stereotaxic Coordinates P21* by Roustem Khazipov et al. was used for P24 scans. *The Rat Brain in Stereotaxic Coordinates* by George Paxinos and Charles Watson (2007) was referenced for P60 animal scans. All segmentations were traced along the coronal plane, consistent with the reference atlases (Figure 1). Scans were randomly assigned to each tracer, and all tracers were blind to treatment groups.

### 2.9. Statistical Analyses

Volumetric data were analyzed using a two-way ANOVA, with inoculation as one factor (three levels: mock, ZIKV, and iZIKV) and sex as another factor (two levels: males and females). For the whole-brain analysis, absolute (non-normalized) volumes were expressed in mm^3^ for base comparison. For the ROI analyses, volumes were expressed as percentage of whole brain (normalized) to control brain size variability. The ROIs included in the automated segmentation analysis were the hippocampus, isocortex, and cerebellum. The ROIs included in the manual segmentation analysis were the whole hippocampus, CA1, CA2, CA3, dentate gyrus, isocortex, and cerebellum. Post-hoc pairwise comparisons were carried out using Tukey’s test (*p* < 0.05) to analyze volumetric differences between inoculation groups. To estimate the degree of agreement between volumes obtained with automated methods and those delineated manually, Pearson’s correlation coefficients were calculated for each brain region of interest at each age point. In addition, Bland–Altman plots were compiled to illustrate the magnitude of the differences between both methods of segmentation. These comparative methods were performed for the regions that overlapped in both segmentation methods: the hippocampus, cerebellum, isocortex, and whole brain.

## 3. Results

### 3.1. Volumetric Analysis of Whole-Brain Volumes Throughout Development Utilizing Two Separate Methods of Segmentation

Absolute volumes for whole-brain (WB) volume were calculated by multiplying the number of the voxels belonging to the structure by voxel volume (mm^3^). Automated segmented WB volumes were calculated by summing up all 26 ROI volumes within the MRH atlas that collectively represent the entire brain [20]. Manual WB volumes were calculated by tracing the outline of the brain in each slice and computing the voxels within the outlined region. Results from this section are summarized in Table S1 which lists mean ± SD of whole-brain volumes for each cohort throughout development.

#### 3.1.1. Postnatal Day 2

**Figure 2:**
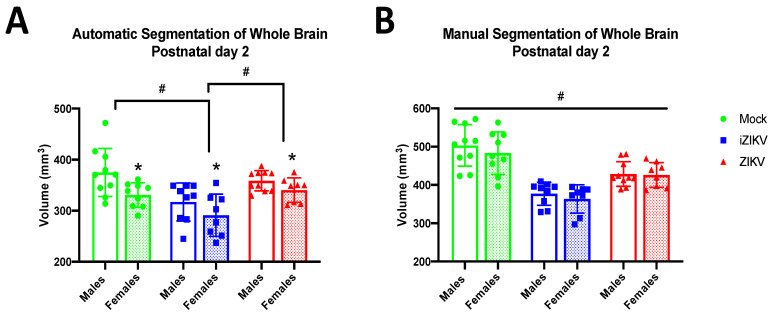
Absolute whole-brain volumes in postnatal day 2 neonates across all three groups stratified by sex using both automated and manual methods of segmentation (*n* = 8–10 per sex per group). (**A**) Automated segmentation revealed a main effect of inoculation (^#^ *p*  <  0.05). Specifically, whole-brain volume was significantly smaller in iZIKV pups when compared to both mock control and ZIKV pups (post-hoc, *^#^ p*  <  0.05). Analysis also revealed a main effect of sex (* *p*  <  0.05); P2 females across all groups had significantly smaller whole-brain volumes than their male counterparts. No interaction between inoculation and sex was detected. (**B**) Manual segmentation also revealed a main effect of inoculation (^#^ *p * <  0.05). iZIKV pups again displayed significantly reduced whole-brain volumes when compared with all other groups; however, ZIKV pups also displayed significantly reduced whole-brain volumes when compared to mock controls (post-hoc, *^#^ p*  <  0.05). There was no main effect of sex or interaction of inoculation × sex. Bars represent the mean ± SD and data points represent individual subjects. Data were analyzed using a two-way ANOVA with inoculation (^#^ *p * <  0.05) and sex (* *p * <  0.05) as factors, followed by Tukey’s test.

At P2, we found a significant effect of inoculation on WB volume in the automated segmentation method (*F*_2,48_ = 11.24, *p* < 0.001; Figure 2A), and post-hoc tests revealed that the iZIKV-inoculated rats had significantly reduced WB volume when compared to the mock and ZIKV cohorts (*p* ≤ 0.001; Figure 2A). We also observed this same phenomenon in the manual segmentation method, where analysis demonstrated significant effects of inoculation (*F*_2,48_ = 38.58, *p* < 0.001; Figure 2B) and further post-hoc tests revealed the iZIKV-inoculated rats had significantly reduced WB volume when compared to both the mock and ZIKV cohorts (*p* ≤ 0.001; Figure 2B). Analysis revealed a main effect of sex for the automated segmentation method (*F*_1,48_ = 10.120, *p* = 0.003; Figure 2A) but not the manual segmentation method (*F*_1,48_ = 1.086, *p* = 0.302; Figure 2B). Furthermore, this analysis revealed no significant interaction effect of inoculation and sex on WB volume in the automated segmentation method (*F*_2,48_ = 0.716, *p* = 0.494) or the manual segmentation method (*F*_2,48_ = 0.203, *p* = 0.817). Thus, both segmentation methods revealed a reduction in iZIKV WB volume.

#### 3.1.2. Postnatal Day 16

**Figure 3:**
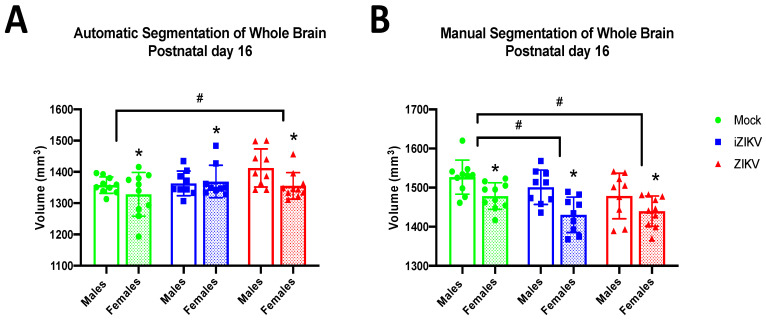
Absolute whole-brain volumes in postnatal day 16 juveniles across all three groups stratified by sex using both automated and manual methods of segmentation (*n* = 9–10 per sex per group). (**A**) Automated segmentation revealed a main effect of inoculation (^#^ *p* < 0.05). Specifically, whole-brain volume was significantly larger in ZIKV juveniles when compared to the mock control group (post-hoc, *^#^ p* < 0.05). (**B**) Manual segmentation also revealed a main effect of inoculation (^#^ *p* < 0.05). Both iZIKV and ZIKV juveniles displayed significantly reduced whole-brain volumes when compared to mock control juveniles (post-hoc, *^#^ p < 0.05*). Analyses also revealed a main effect of sex for both segmentation methods (* *p* < 0.05). More specifically, P16 females across all groups had significantly smaller whole-brain volumes than their male counterparts. There was no interaction of inoculation × sex within either method of segmentation. Bars represent the mean ± SD and data points represent individual subjects. Data were analyzed using a two-way ANOVA with inoculation (^#^ *p* < 0.05) and sex (* *p* < 0.05) as factors, followed by Tukey’s test.

At P16, we found a significant effect of inoculation on WB volume in both the automated segmentation method (*F*_2,51_ = 3.19, *p* = 0.049; Figure 3A) and the manual segmentation method (*F*_2,51_ = 5.37, *p* = 0.008; Figure 3B). Further analysis with post-hoc tests revealed that ZIKV WB volumes were significantly larger than their mock counterparts (*p* = 0.049; Figure 3A) when WB volumes were collected using the automated segmentation method. In contrast, post-hoc tests for the manually segmented WB volumes revealed iZIKV (*p* = 0.037) and ZIKV (*p* = 0.009; Figure 3B) volumes were significantly smaller than mock WB volumes. Analysis revealed a significant effect of sex on WB volumes at this age for both the automated segmentation method (*F*_1,51_ = 4.03, *p =* 0.50; Figure 3A) and manual segmentation method (*F*_1,51_ = 20.04, *p <* 0.001; Figure 3B). As expected, WB volumes in P16 males are significantly larger than those in P16 females. However, no significant interaction effect of inoculation and sex on WB volume was observed in the automated segmentation method (*F*_2,51_ = 1.83, *p* = 0.171) or the manual segmentation method (*F*_2,51_ = 0.62, *p* = 0.542). 

#### 3.1.3. Postnatal Day 24

**Figure 4:**
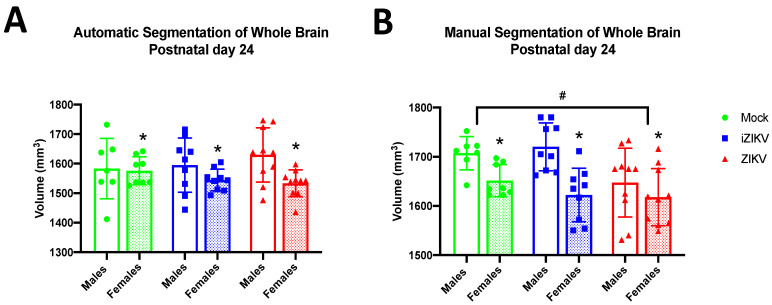
Absolute whole-brain volumes in postnatal day 24 juveniles across all three groups stratified by sex using both methods of segmentation (*n* = 7–10 per sex per group). (**A**) Automated segmentation revealed no main effect of inoculation. (**B**) Manual segmentation revealed a main effect of inoculation (^#^ *p*  <  0.05). ZIKV juveniles displayed significantly reduced whole-brain volumes when compared to mock control juveniles (post-hoc, *^#^ p*  <  0.05). Analyses also revealed a main effect of sex for both segmentation methods (* *p*  <  0.05). More specifically, P24 females across all groups had significantly smaller whole-brain volumes than their male counterparts. There was no interaction of inoculation × sex within either method of segmentation. Bars represent the mean  ±  SD and data points represent individual subjects. Data were analyzed using a two-way ANOVA with inoculation (^#^ *p*  <  0.05) and sex (* *p*  <  0.05) as factors, followed by Tukey’s test.

At P24, we found a significant effect of inoculation on WB volume through our manual segmentation method (*F*_2,47_ = 4.10, *p* = 0.023; Figure 4B). Post-hoc tests revealed that WB volumes in the ZIKV-inoculated cohort were significantly smaller in comparison to the mock-inoculated cohort (*p* = 0.042; Figure 4B) at the P24 age point. In contrast, there was no significant effect of inoculation on WB volume in the automated results (*F*_2,47_ = 0.14, *p* = 0.872; Figure 4A). The significant effect of sex on WB volumes seen at P16 was maintained for P24 for both the automated segmentation method (*F*_1,47_ = 6.49, *p =* 0.014; Figure 4A) and manual segmentation method (*F*_1,47_ = 17.41, *p <* 0.001; Figure 4B). Furthermore, analysis revealed no significant interaction between inoculation and sex in the automated segmentation method (*F*_2,47_ = 4.10, *p* = 0.023) or the manual segmentation method (*F*_2,47_ = 1.60, *p* = 0.214). 

#### 3.1.4. Postnatal Day 60

**Figure 5:**
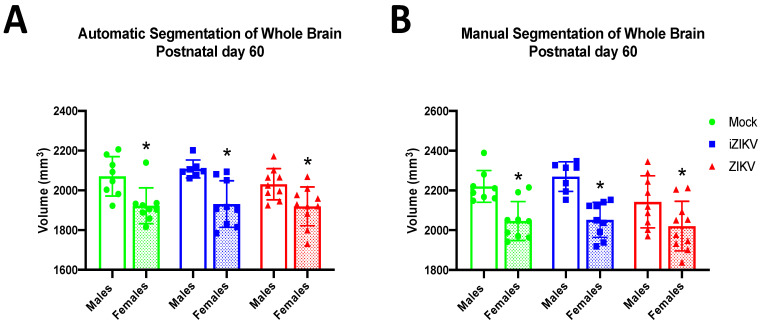
Absolute whole-brain volumes in postnatal day 60 adults across all three groups stratified by sex using both automated segmentation (**A**) and manual segmentation (**B**), (*n* = 7–10 per sex per group). Neither method detected a main effect of inoculation. Within both segmentation methods, analyses revealed a main effect of sex (* *p*  <  0.05). More specifically, P60 females across all groups had significantly smaller whole-brain volumes than their male counterparts. There was no interaction of inoculation × sex within either method of segmentation. Bars represent the mean  ±  SD and data points represent individual subjects. Data were analyzed using a two-way ANOVA with inoculation and sex (* *p* < 0.05) as factors, followed by Tukey’s test.

At P60, we found no significant effect of inoculation on WB volume in either segmentation method. Thus, both the automated segmentation method (*F*_2,46_ = 0.99, *p* = 0.3791; Figure 5A) and manual segmentation method (*F*_2,46_ = 2.65, *p* = 0.081; Figure 5B) demonstrate that there are no WB volume differences between cohorts as a result of inoculation. More interestingly, these data seem to suggest that the effects of inoculation on WB volume seen in early age points have been somehow ameliorated. As seen at most age points, analysis revealed a significant effect of sex on WB volume in both segmentation methods (automated: *F*_2,46_ = 31.86, *p* < 0.001, manual: *F*_2,46_ = 34.90, *p* < 0.001; Figure 5A,B), which is line with previous literature that demonstrates substantial sex difference in rodent brain size. Moreover, we saw no significant interaction of inoculation and sex in either the automated (*F*_2,46_ = 0.54, *p* = 0.584) or manual (*F*_2,46_ = 0.92, *p* = 0.408) segmentation method.

### 3.2. Volumetric Analysis of Regional Brain Volumes Throughout Development Utilizing Two Separate Methods of Segmentation

Absolute volumes for each brain region were calculated in the same manner as whole-brain volumes described above. Automated ROI volumes were calculated by extracting the boundary of each individual ROI after properly registering the MR image to the MR atlas. Manual ROI volumes were calculated by tracing each individual brain structure and then multiplying the number of the voxels belonging to the structure by voxel volume (mm^3^). Regional brain volume differences were then corrected by dividing structure volume by whole-brain volume for each rat. ROI volumes were normalized in this manner to account for large brain variabilities seen among individual rats. Data from this section are summarized in Table S2, which lists mean ± SD of all regional brain volumes analyzed for each cohort throughout development.

#### 3.2.1. Automated Segmentation at Postnatal Day 2

**Figure 6:**
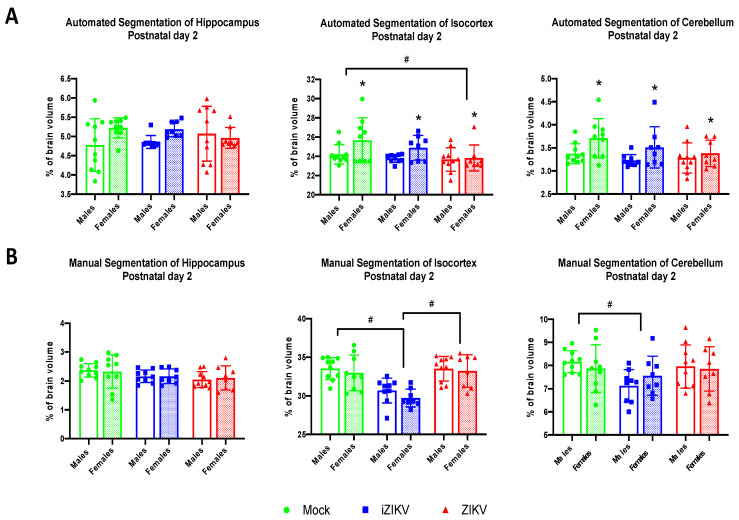
(**A**) Automated segmentation of brain regions at postnatal day 2 revealed a significant effect of inoculation. Analysis revealed ZIKV-inoculated neonates had a smaller isocortex when compared to mock-inoculated neonates (post-hoc,*^#^ p* < 0.05). Furthermore, analysis revealed a significant effect of sex in both the isocortex and cerebellum (* p < 0.05). Analysis detected no interaction of inoculation × sex. (**B**) Manual segmentation of brain regions at postnatal day 2 revealed a significant effect of inoculation. Within the isocortex, iZIKV-inoculated neonates displayed smaller regional volumes when compared to both mock and ZIKV counterparts (post-hoc,*^#^ p* < 0.05). Analysis also revealed iZIKV neonates had smaller cerebellums in comparison to the mock controls (post-hoc,*^#^ p* < 0.05). Analysis detected no significant effect of sex or interaction of inoculation × sex. Bars represent the mean ± SD and data points represent individual subjects (*n* = 8–10 per sex per group). Data were analyzed using a two-way ANOVA with inoculation (*^#^ p* < 0.05) and sex (* p < 0.05) as factors, followed by Tukey’s test.

Automated segmentation at the P2 age point revealed a significant effect of inoculation within the CTX (*F*_2,48_ = 3.37, *p* = 0.043; Figure 6A). Post-hoc tests revealed that the ZIKV cohort had reduced cortical volumes when compared to the mock cohort (*p* = 0.038; Figure 6A). Furthermore, we found no significant effect of inoculation with the HC (*F*_2,48_ = 0.01, *p* = 0.988) or the CB (*F*_2,48_ = 2.18, *p* = 0.125; Figure 6A). 

Interestingly, we also found a significant effect of sex within the CTX (*F*_1,48_ = 5.74, *p* = 0.021) and the CB (*F*_1,48_ = 7.53, *p* = 0.009; Figure 6A). Males were shown to have reduced cortical volumes when compared to females. This sex difference was also maintained within the CB, where we observed reduced cerebellar volumes in males when compared to females (Figure 6A). There was no significant effect of sex within the HC (*F*_1,48_ = 3.08, *p* = 0.086). Moreover, analysis revealed no significant interaction of inoculation and sex within any of the brain regions: HC (*F*_2,48_ = 1.18, *p* = 0.175), CTX (*F*_2,48_ = 1.07, *p* = 0.352), and CB (*F*_2,48_ = 0.68, *p* = 0.521). 

#### 3.2.2. Manual Segmentation at Postnatal Day 2

Regional brain volumes extracted via manual segmentation revealed that a significant effect of inoculation within the CTX (*F*_2,48_ = 18.65, *p* < 0.001) and CB (*F*_2,48_ = 0.002, *p* = 0.047; Figure 6B). Post-hoc tests revealed that iZIKV-inoculated neonates had significantly less cortical volume than both their mock-inoculated and ZIKV-inoculated counterparts (*p* < 0.001; Figure 6B). Within the CB, post-hoc tests revealed that the iZIKV cohort also had significantly less cerebellar volume when compared to the mock cohort (*p* = 0.043). There was no main effect of inoculation on HC volumes (*F*_2,48_ = 2.87, *p* = 0.066; Figure 6B)

At P2, we found no significant effect of sex on any of the brain regions analyzed: HC (*F*_1,48_ =0.004, *p* = 0.948), CTX (*F*_1,48_ = 1.67, *p* = 0.203), and CB (*F*_1,48_ = 0.002, *p* = 0.966) and furthermore no interaction of inoculation and sex on any of the brain regions analyzed: HC (*F*_2,48_ =0.84, *p* = 0.920), CTX (*F*_2,48_ = 0.17 *p* = 0.845, and CB (*F*_2,48_ = 0.89, *p* = 0.416). Overall, these data indicate regional volume reductions within the iZIKV group at P2 independent of sex. 

#### 3.2.3. Automated Segmentation at Postnatal Day 16

**Figure 7:**
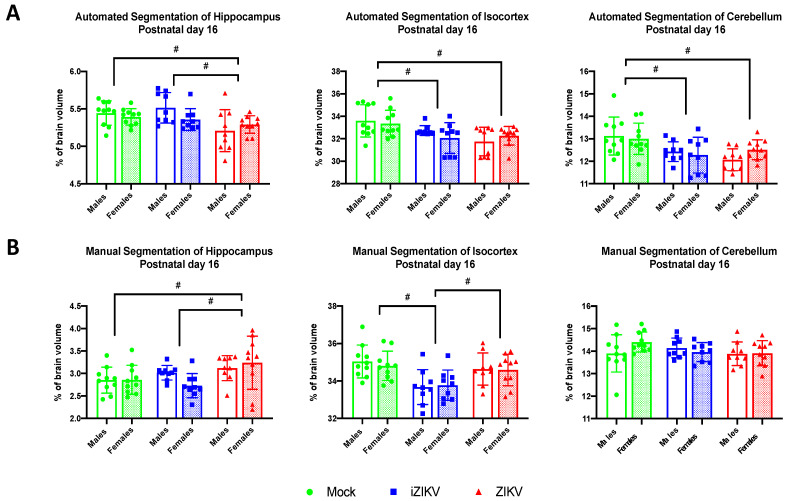
(**A**) Automated segmentation of brain regions at postnatal day 16 revealed a significant effect of inoculation. Within the hippocampus region, ZIKV-inoculated juveniles displayed smaller regional volumes when compared to both mock and iZIKV counterparts (post-hoc,*^#^ p* < 0.05). Within the isocortex and cerebellum, both iZIKV-inoculated and ZIKV-inoculated juveniles displayed smaller regional volumes when compared to mock controls (post-hoc,*^#^ p* < 0.05). Analysis detected no significant effect of sex or interaction of inoculation × sex. (**B**) Manual segmentation of brain regions at postnatal day 16 revealed a significant effect of inoculation. Within the hippocampus region, ZIKV-inoculated juveniles displayed larger regional volumes when compared to both mock and iZIKV counterparts (post-hoc,*^#^ p* < 0.05). Within the isocortex, iZIKV-inoculated juveniles displayed smaller regional volumes when compared to both mock and ZIKV counterparts (post-hoc, *^#^ p* < 0.05). Analysis detected no significant effect of sex or interaction of inoculation × sex. Bars represent the mean ± SD and data points represent individual subjects (*n* = 9–10 per sex per group). Data were analyzed using a two-way ANOVA with inoculation (*^#^ p* < 0.05) and sex as factors, followed by Tukey’s test.

At P16, automated segmentation revealed a main effect of inoculation on all brain regions that were analyzed: HC (*F*_2,51_ =6.37, *p* = 0.003), CTX (*F*_2,51_ =8.55, *p* = 0.001), and CB (*F*_2,51_ = 8.67, *p* = 0.001; Figure 7A). Post-hoc analyses revealed that ZIKV-inoculated rats had significantly reduced hippocampal volumes at P16 when compared to the mock control group and the iZIKV group (*p* = 0.014; Figure 7A). Interestingly, this volume reduction within the hippocampus did not appear at the earlier P2 age point in either segmentation method. Within the CTX, post-hoc tests demonstrated that both iZIKV rats (*p* = 0.015) and ZIKV rats (*p* = 0.001; Figure 7A) had reduced cortical volumes when compared to the control group. A similar effect was revealed through post-hoc analysis of the cerebellar region at P16. Both iZIKV (*p* = 0.004) and ZIKV (*p* = 0.002; Figure 7A) cerebellums were smaller than mock cerebellums during juvenile development. 

We found no significant effect of sex on any of the brain regions analyzed: HC (*F*_1,51_ = 0.83, *p* = 0.374), CTX (*F*_1,51_ = 0.19, *p* = 0.667), and CB (*F*_1,51_ = 0.12, *p* = 0.733) and furthermore no interaction of inoculation and sex on any of the brain regions analyzed: HC (*F*_2,51_ = 2.10, *p* = 0.133), CTX (*F*_2,51_ = 1.26, *p* = 0.293), and CB (*F*_2,51_ = 0.54, *p* = 0.279). These data reveal that regional volume reductions in the iZIKV and ZIKV cohorts the are independent of sex. 

#### 3.2.4. Manual Segmentation at Postnatal Day 16

Manual segmentation at P16 revealed a main effect of inoculation within the HC region (*F*_2,51_ = 5.12, *p* = 0.009) and CTX region (*F*_2,51_ = 10.04, *p* < 0.001; Figure 7B). In contrast to the results derived from the automated segmentation, post-hoc tests revealed that ZIKV-inoculated juveniles displayed increased hippocampal volumes in comparison to both mock (*p* = 0.015) and iZIKV (*p* = 0.026; Figure 7B) juveniles. Within the CTX post-hoc tests, we found that iZIKV juveniles had reduced cortex volumes when compared to both mock (*p* < 0.001) and ZIKV (*p* = 0.007; Figure 7B) juveniles. We found no main effect of inoculation within the CB region (*F*_2,51_ = 1.05, *p* = 0.359; Figure 7B)

We found no significant effect of sex on any of the brain regions analyzed: HC (*F*_1,51_ = 0.32, *p* = 0.572), CTX (*F*_1,51_ = 0.71, *p* = 0.791), and CB (*F*_1,51_ = 0.61, *p* = 0.439) and furthermore no interaction of inoculation and sex on any of the brain regions analyzed: HC (*F*_2,51_ = 1.64, *p* = 0.204), CTX (*F*_2,51_ = 0.18, *p* = 0.836), and CB (*F*_2,51_ = 1.93, *p* = 0.155). Overall, these data indicate that regional volume reductions within observed iZIKV and ZIKV juveniles are independent of sex. 

#### 3.2.5. Automated Segmentation at Postnatal Day 24

**Figure 8:**
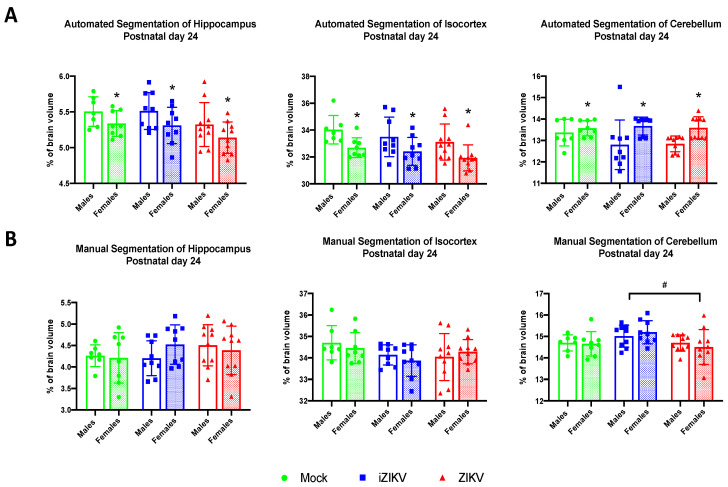
(**A**) Automated segmentation of brain regions for postnatal day 24 revealed a main effect of sex in all regions analyzed (* p < 0.05). Furthermore, analysis identified no significant effect of inoculation or interaction of inoculation × sex. (**B**) Manual segmentation of brain regions at postnatal day 24 revealed a significant effect of inoculation. Within the cerebellum region, ZIKV-inoculated juveniles displayed smaller regional volumes when compared to the iZIKV-inoculated juveniles (post-hoc,*^#^ p* < 0.05). Analysis detected no significant effect of sex or interaction of inoculation × sex. Bars represent the mean ± SD and data points represent individual subjects (*n* = 7–10 per sex per group). Data were analyzed using a two-way ANOVA with inoculation (*^#^ p* < 0.05) and sex (* p < 0.05) as factors, followed by Tukey’s test.

Analysis of regional brain volumes at P24 revealed no significant effect of inoculation in any of the brain regions: HC (*F*_2,47_ = 3.52, *p* = 0.060), CTX (*F*_2,47_ = 2.33, *p* = 0.109, and CB (*F*_2,47_ = 0.80, *p* = 0.454; Figure 8A). There was, however, a significant effect of sex within the HC (*F*_1,47_ = 7.41, *p* = 0.009), CTX (*F*_1,47_ = 14.53, *p* < 0.001), and CB (*F*_1,47_ = 12.10, *p* = 0.001). Males demonstrated larger HC and CTX volumes while females demonstrated larger CB volume. There was no significant no interaction of inoculation and sex on any of the brain regions analyzed: HC (*F*_2,47_ = 0.21, *p* = 0.979), CTX (*F*_2,47_ = 0.05, *p* = 0.950), and CB (*F*_2,47_ = 1.27, *p* = 0.289). 

#### 3.2.6. Manual Segmentation at Postnatal Day 24

Manual segmentation at the P24 age point revealed a significant effect of inoculation in the CB region (*F*_2,47_ = 4.23, *p* = 0.021; Figure 8B). Post-hoc tests revealed increased CB volumes in the iZIKV group when compared to the ZIKV group (*p* = 0.023; Figure 8B). There was no significant effect of inoculation on HC volumes (*F*_2,47_ = 0.82, *p* = 0.446) or CTX volumes (*F*_2,47_ = 2.41, *p* = 0.100; Figure 8B). 

We found no significant effect of sex on any of the brain regions analyzed: HC (*F*_1,47_ = 0.15, *p* = 0.705), CTX (*F*_1,47_ = 0.20, *p* = 0.658), and CB (*F*_1,47_ = 0.009, *p* = 0.924) and furthermore no interaction of inoculation and sex on any of the brain regions analyzed: HC (*F*_2,47_ = 1.10, *p* = 0.340), CTX (*F*_2,47_ = 0.67, *p* = 0.100), and CB (*F*_2,47_ = 0.53, *p* = 0.592). Overall, these data indicate the regional volume differences observed are independent of sex. 

#### 3.2.7. Automated Segmentation at Postnatal Day 60

**Figure 9:**
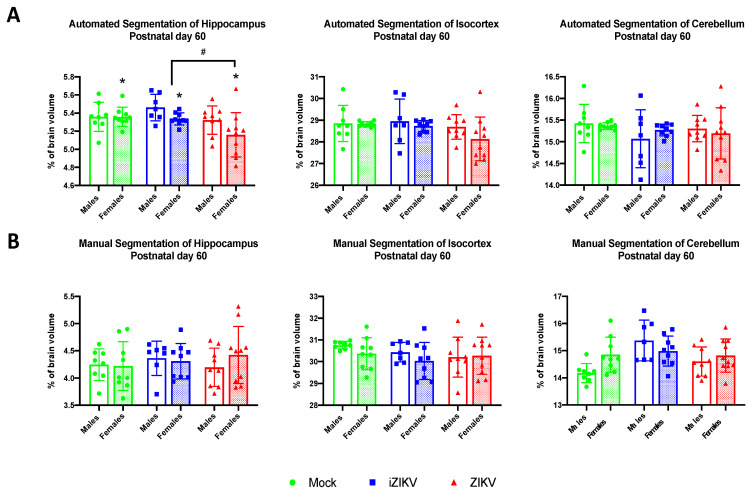
(**A**) Automated segmentation of brain regions at postnatal day 60 revealed a significant effect of inoculation. Within the hippocampus region, ZIKV-inoculated juveniles displayed smaller regional volumes when compared to the iZIKV-inoculated juveniles (post-hoc,*^#^ p* < 0.05). Moreover, analysis also revealed a main effect of sex within the hippocampal region (* p < 0.05). Analysis detected no interaction of inoculation × sex. (**B**) Analysis of manually segmented brain regions for postnatal day 60 revealed no significant effect of inoculation, sex, or interaction of inoculation × sex. Bars represent the mean ± SD and data points represent individual subjects (*n* = 7–10 per sex per group). Data were analyzed using a two-way ANOVA with inoculation (*^#^ p* < 0.05) and sex (* p < 0.05) as factors, followed by Tukey’s test.

At P60, analysis revealed a significant effect of inoculation in the HC region (*F*_2,46_ = 4.68, *p* = 0.037; Figure 9A). Post-hoc analysis revealed reduced HC volume in ZIKV-inoculated adults when compared to the iZIKV group (*p* = 0.018; Figure 9A). We also observed a significant effect of sex in the HC region (*F*_1,46_ = 4.64, *p* = 0.037; Figure 9A), with females demonstrating smaller HC volumes than males. There was no main effect of inoculation within the CTX (*F*_2,46_ = 2.13, *p* = 0.131) or the CB (*F*_2,46_ = 1.19, *p* = 0.313; Figure 9A) and no main effect of sex in the CTX (*F*_1,46_ = 1.79, *p* = 0.187 or the CB (*F*_1,46_ = 0.01, *p* = 0.922). We found no significant no interaction of inoculation and sex on any of the brain regions analyzed: HC (*F*_2,46_ = 1.24, *p* = 0.298), CTX (*F*_2,46_ = 0.62, *p* = 0.544), and CB (*F*_2,46_ = 0.67, *p* = 0.516). 

#### 3.2.8. Manual Segmentation at Postnatal Day 60

We found no significant effect of inoculation on any of the brain regions analyzed: HC (*F*_2,46_ = 0.32, *p* = 0.730), CTX (*F*_2,46_ = 0.65, *p* = 0.525), and CB (*F*_2,46_ = 1.73, *p* = 0.189; Figure 9B); no significant effect of sex any of the brain regions analyzed: HC (*F*_1,46_ = 0.20, *p* = 0.656), CTX (*F*_1,46_ = 2.11, *p* = 0.153), and CB (*F*_1,46_ = 1.45, *p* = 0.234); and furthermore no interaction of inoculation and sex on any of the brain regions analyzed: HC (*F*_2,46_ = 0.71, *p* = 0.499), CTX (*F*_2,46_ = 0.34, *p* = 0.712), and CB (*F*_2,46_ = 1.23, *p* = 0.301). Taken together these data suggest that during neurodevelopment, any regional undergrowth or overgrowth observed as a result of inoculation disappears later in adulthood.

### 3.3. Volumetric Analysis of Manually Segmented Hippocampal Subregions Throughout Development

In addition to whole hippocampal volumes, we analyzed individual subregions of the hippocampus, including the CA1, CA2, CA3, and dentate gyrus, across all ages for any volumetric differences between the diluent, ZIKV, and iZIKV groups (Table S3). Hippocampal subregions were traced manually and calculated similarly to the method described above.

#### 3.3.1. Postnatal Day 2

**Figure 10:**
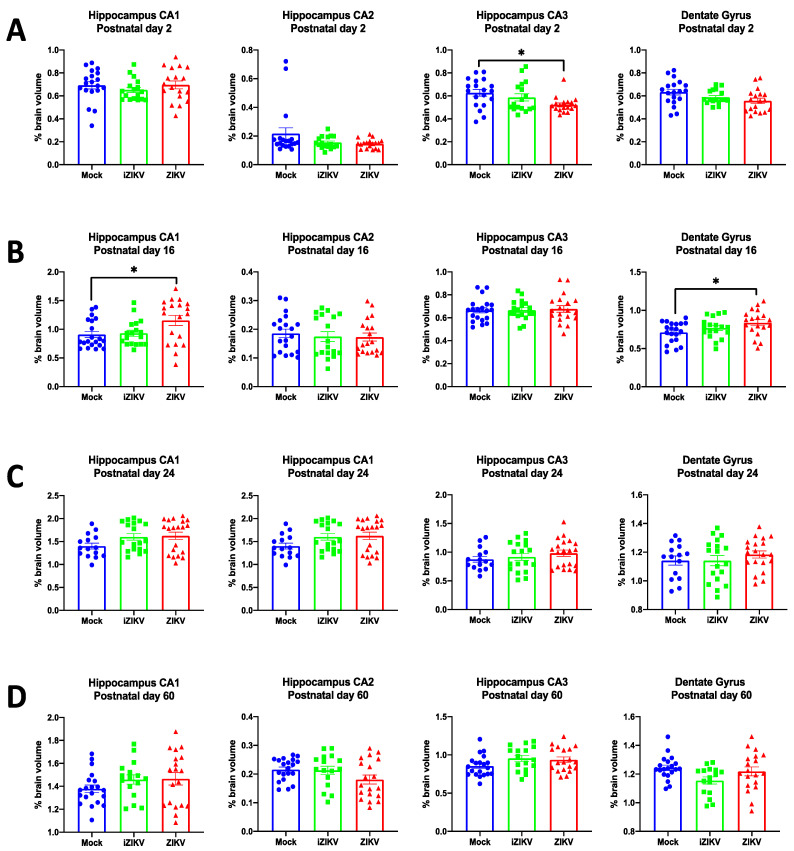
Analysis of manually segmented hippocampal subregions for postnatal day 2 (*n* = 17–19 per group) (**A**), postnatal day 16 (*n* = 18–20 per group) (**B**), postnatal day 24 (*n* = 15–20 per group) (**C**), and postnatal day 60 (*n* = 16–19 per group) (**D**). At P2, volumetric analysis of hippocampal subregions revealed a significant effect of inoculation within the CA3 of the hippocampus. Specifically, the ZIKV-inoculated pups displayed a significant volume reduction in the CA3 subregion when compared to mock counterparts (post-hoc, * p < 0.05). At P16, analysis revealed a significant effect of inoculation within the CA1 of the hippocampus and dentate gyrus. Specifically, ZIKV-inoculated juveniles displayed significantly larger volumes in both the CA1 subregion and dentate gyrus when compared to mock controls (post-hoc, * p < 0.05*)*. Across all groups, bars represent the mean ± SD in each subregion of the hippocampus while data points represent each individual rat.

At P2, manual segmentation revealed a significant effect of inoculation for the CA3 (*F*_2,48_ = 3.92, *p* = 0.027; Figure 10A). Within CA3 post-hoc tests, we found ZIKV rats had smaller CA3 volumes when compared to mock rats (*p* = 0.019; Figure 10A). We did not find any significant effect of inoculation in the CA1 (*F*_2,48_ = 1.05, *p* = 0.358), CA2 (*F*_2,48_ = 2.12, *p* = 0.132), or DG (*F*_2,48_ = 2.78, *p* = 0.072; Figure 10A). Furthermore, we observed no sex differences (CA1: *F*_1,48_ = 0.27, *p* = 0.603; CA2: *F*_1,48_ = 0.45, *p* = 0.504; CA3: *F*_1,48_ = 0.19, *p* = 0.664; DG: *F*_1,48_ = 0.11, *p* = 0.737) or interaction of inoculation and sex (CA1: *F*_2,48_ = 0.30, *p* = 0.744; CA2: *F*_2,48_ *=* 1.00, *p* = 0.375 CA3: *F*_2,48_ *=* 1.06, *p* = 0.354; DG: *F*_2,48_ *=* 0.10, *p* = 0.906) for any of the hippocampal subregions.

#### 3.3.2. Postnatal Day 16

Analysis of the hippocampal subregions at P16 demonstrated a significant effect of inoculation for the CA1 subregion (*F*_2,51_ = 3.84, *p* = 0.028) and the DG subregion (*F*_2,51_ = 3.67, *p* = 0.033; Figure 10B). Interestingly, post-hoc tests revealed that the ZIKV-inoculated cohort had larger CA1 (*p* = 0.037) and DG (*p* = 0.023; Figure 10B) volumes than their mock-inoculated counterparts. We did not find any significant effect of inoculation in the CA2 (*F*_2,48_ = 0.22, *p* = 0.801) or CA3 (*F*_2,48_ = 0.09, *p* = 0.916; Figure 10B). Moreover, there was no significant effect of sex (CA1: *F*_1,51_ = 0.10, *p* = 0.919; CA2: *F*_1,51_ = 0.003, *p* = 0.958; CA3: *F*_1,51_ = 0.60, *p* = 0.442; DG: *F*_1,51_ = 1.08, *p* = 0.304) or interaction of inoculation and sex (CA1: *F*_2,51_ = 0.01, *p* = 0.919; CA2: *F*_2,51_ *=* 1.26, *p* = 0.293; CA3: *F*_2,51_ *=* 0.12, *p* = 0.895; DG: *F*_2,51_ *=* 2.31, *p* = 0.110) for any of the hippocampal subregions.

#### 3.3.3. Postnatal Day 24

At P24, we found no significant effect of inoculation (CA1: *F*_2,47_ = 2.42, *p* = 0.100 CA2: *F*_2,47_ *=* 2.32 *p* = 0.110; CA3: *F*_2,47_ *=* 0.86, *p* = 0.429; DG: *F*_2,47_ *=* 0.63, *p* = 0.537; Figure 10C), no sex differences (CA1: *F*_1,47_ = 0.19, *p* = 0.662; CA2: *F*_1,47_ = 0.70, *p* = 0.406; CA3: *F*_1,47_ = 0.13, *p* = 0.722; DG: *F*_1,47_ = 0.80, *p* = 0.376), and no significant interaction of inoculation and sex (CA1: *F*_2,47_ = 0.35, *p* = 0.704; CA2: *F*_2,47_ *=* 0.30, *p* = 0.740; CA3: *F*_2,47_ *=* 0.63, *p* = 0.537; DG: *F*_2,47_ *=* 0.65, *p* = 0.937) for any of the hippocampal subregions.

#### 3.3.4. Postnatal Day 60

The results from P24 were maintained at P60 age point, and we found no significant effect of inoculation (CA1: *F*_2,49_ = 1.26, *p* = 0.292; CA2: *F*_2,49_ *=* 2.22, *p* = 0.120; CA3: *F*_2,49_ *=* 2.13, *p* = 0.130; DG: *F*_2,49_ *=* 2.94, *p* = 0.062; Figure 10D), no significant no sex differences (CA1: *F*_2,49_ < 0.01, *p* = 0.985; CA2: *F*_1,49_ = 1.49, *p* = 0.228; CA3: *F*_1,49_ = 1.18, *p* = 0.283; DG: *F*_1,49_ = 2.58, *p* = 0.115), and no significant interaction of inoculation and sex (CA1: *F*_2,49_ = 0.56, *p* = 0.945; CA2: *F*_2,49_ *=* 0.16, *p* = 0.857; CA3: *F*_2,49_ *=* 2.06, *p* = 0.139; DG: *F*_2,49_ *=* 1.67, *p* = 0.329) for any of the hippocampal subregions.

### 3.4. Automated Versus Manual Segmentation of Brain Region Volumes

#### 3.4.1. Pearson Correlations

Pearson correlations were calculated to assess the degree of agreement between volumes obtained via the automated segmentation method and the manual segmentation method. Raw values extracted from both the automated segmentation and manual segmentation were compared for the cerebellum (CB), cortex (CTX), hippocampus (HC), and whole brain (WB) regions at each of the four age points. Agreement between automated and manual segmentation varied based on age point and brain region. Pearson’s correlations revealed significantly positive slopes in all brain regions analyzed at the P2 age point, suggesting moderate to strong agreement between the two approaches (range: 0.38–0.79; *p* < 0.05, corrected) (Table S4). At the P16 age point, correlations between manual segmentation and automated methods were strongest in the CB region (*r* = 0.43, *p* < 0.05) and the CTX region (*r* = 0.62, *p* < 0.05) (Table S4). The P24 age point revealed moderate agreement between the two approaches in the CTX region (*r* = 0.35, *p* < 0.05) (Table S4). At the P60 age point, we again observed significantly positive slopes in all brain regions analyzed, suggesting moderate to strong agreement between the two methods at this age (*r* range: 0.36–0.59; *p* < 0.05, corrected) (Table S4).

#### 3.4.2. Bland–Altman Plots

Bland–Altman plots were compiled to investigate any systematic biases between the two segmentation methods used. It was our interest to determine if one method consistently overestimates or underestimates volume more than the other [21]. Specifically, these plots were constructed by computing the volumetric difference between manual segmentation and automated methods for each structure of interest. The Bland–Altman approach suggests that these two methods are not interchangeable even when the Pearson correlations show strong to moderate agreement. The plots revealed proportional biases indicating that the methods do not agree equally (Figure S1A–D). The automated segmentation method frequently underestimated WB, CB, and CTX volumes. Interestingly, when analyzed with the Bland–Altman approach, HC volumes were overestimated for all age points by the automated segmentation method. Taken together, these data suggest that small-volume structures such as the HC tended to be “overestimated” across all age points by the automated segmentation method while large-volume structures such as the WB, CB, and CTX tended to be “underestimated” across all age points by the automated segmentation method.

## 4. Discussion

This study is the first of its kind to utilize a longitudinal in vivo MRI technique with a dual approach of automated segmentation and manual segmentation in order to explore the neurodevelopmental outcomes and potential neurological sequelae of prenatal ZIKV infection in rodent offspring. While comparative analysis revealed relatively low agreement between our segmentation methods, the study presents significant novel findings derived from the manual segmentation, which is regarded as the “gold standard” method for quantifying volumetric differences among brain structures [22,23,24]. First, we observed sex differences in whole-brain volumes which emerged after the P2 age point and continued into adulthood (P60). This is to be expected, with male rats having larger brains on average than females. Furthermore, we observed no sex differences in any of the individual brain regions analyzed throughout P2–P60, which demonstrates the effectiveness and importance of normalizing the volume of individual brain regions to the total brain volume for understanding true sex differences in the size of individual structures. More importantly, we found no significant interaction of sex and inoculation in any brain region during all four age points. These data indicate that the effect of inoculation is independent of sex for all regions analyzed in the current study. Furthermore, our results demonstrate prenatal ZIKV infection results in profound alterations of brain development in affected offspring. In particular, our study illustrates that prenatal ZIKV infection significantly impacts brain growth of affected offspring at different developmental milestones in both the whole brain and hippocampus. Notably, we find that prenatal ZIKV infection results in transient increases and decreases in hippocampal subregions during the neonatal and juvenile developmental time periods. Moreover, we also report significant volume reductions in the whole brain, cerebellum, and cortex in our iZIKV cohort during the neonatal and juvenile age points. These data suggest the presence of viral particles may stimulate a robust maternal innate immune response, which has been linked to perturbation of brain development mechanisms in affected offspring [25,26,27]. Most interestingly, the findings in both the ZIKV and iZIKV cohorts disappear by adulthood. At P60, we find that there are no significant volumetric differences in the whole brain, cerebellum, cortex, whole hippocampus, or hippocampal subregions between groups. 

Neurodevelopment is an intricately complex process that begins early in gestation and is particularly vulnerable to a variety of insults [28,29]. Substantial evidence has demonstrated that maternal infection can affect critical aspects of fetal brain development during particular periods of heightened susceptibility, leading to a wide range of sequelae such as neuronal dysfunctions and behavioral outcomes in offspring [30]. Similarly, maternal infection with ZIKV is thought to lead to alterations in neurodevelopment through the direct effects of the virus on developing microglia and maternal immune activation itself [31]. However, postviral sequelae associated with prenatal ZIKV infection are not well understood despite considerable research with the use of murine models. Furthermore, epidemiological studies in humans are temporally constrained, currently offering only a snapshot of immediate-term outcomes which have consisted of a wide spectrum of structural and functional abnormalities and impairments [32]. There is, however, a growing body of evidence that suggests ZIKV infection is associated with persistent neurological sequelae. Notably, research has also shown ZIKV infection attenuates the growth of human neural progenitor cells (hNPCs) [33]. These hNPCs not only play critical roles during fetal brain development, but also persist in the adult brain throughout life [34], suggesting that the ZIKV-mediated growth retardation potentially contributes to long-term neurodevelopmental defects through exposure to the virus. Moreover, these studies also suggest ZIKV targets different brain cells throughout the course of brain development [35], preferentially targeting neural progenitor cells that seed and pattern the developing cortex [36]. The neurotropic properties of ZIKV and its impact on developing neural cells raise concerns about the potential long-term neurological sequelae of ZIKV infection. 

The full trajectory of associated consequences as a result of prenatal ZIKV infection is unknown. However, the current findings presented in this study suggest that prenatal ZIKV infection alters the normal course of brain development. We observed significant reductions in whole-brain volume throughout P2–P24 within the ZIKV-inoculated group. Interestingly, this volume deficit was no longer significant at the P60 age point. One possible explanation for this could be that the prenatal ZIKV injury in our model occurs outside the window in which long-standing structural changes become more entrenched and difficult to reverse. A second notable observation from these data is the alterations in growth patterns within the hippocampal subregions. Our results revealed volume reduction in the CA3 subregion at P2 and volume overgrowth in the CA1 and dentate gyrus at P16, which suggest substantially altered developmental trajectory of these hippocampal subregions as a result of prenatal ZIKV infection. By P60 we no longer observed any significant volumetric differences between groups within the hippocampal subregions. The hippocampus is one of the brain regions that grow exponentially during postnatal development in both humans and rodents [37]. This prolonged neural plasticity in the hippocampus may explain why volume alterations observed within these subregions “improve” or normalize by adulthood. It is important to consider volume is not a direct measure of brain function; therefore, our findings warrant further investigation into the potential role of prenatal ZIKV infection in regard to adult brain functionality. Further longitudinal research to rule out possible functional anomalies such as altered brain connectivity, neuronal dysfunction, changes in gene expression profiles, and prolonged cytokine dysregulation would be a significant step in attempting to understanding the full scope of potential long-term neurological sequelae after ZIKV infection. 

As an important control, we inoculated a separate set of pregnant female rats with UV-inactivated ZIKV (iZIKV). Our rationale for including this control is based on epidemiological research indicating maternal immune activation or prenatal infections are considered a risk factor for a number of neurodevelopmental disorders in the offspring, including autism spectrum disorder (ASD), epilepsy, and schizophrenia [25]. Animal models of maternal immune activation have recapitulated altered brain development in offspring as a result of prenatal infections [38,39,40]. Furthermore, maternal immune activation studies focused on long-term sequelae have identified a wide spectrum of abnormalities, such as cognitive and behavioral deficits, present in both adolescent and adult offspring [41]. Despite a growing body of literature, the full impact of maternal immune activation on neuronal development and brain structure in affected progeny is not well understood [42]. Thus, there are a number of laboratories studying the outcomes of maternal immune activation on fetal neurodevelopment [43,44]. By inoculating dams with iZIKV at the same “dose” as our ZIKV inoculations, we sought to test whether the subtler effects of ZIKV infection on the fetal brain throughout development can be replicated by simple maternal immune activation alone and not an active infection transmitted from the dam to the fetus. We find that the iZIKV cohort had significant volume reductions in the whole brain and cortex at P2 through P16. Furthermore, iZIKV neonates also demonstrated smaller cerebellar volumes which later disappeared at the juvenile and adult age points (P16–P60). Moreover, previous MRI studies have identified reductions in the volume of several brain regions in rodent and nonhuman primate offspring as a result of maternal immune activation [45,46,47]. Taken together, one plausible explanation is that maternal immune activation itself is responsible for the structural alterations seen in developing iZIKV offspring via aberrant neural development. Further exploration into the cytokine response after iZIKV inoculation in pregnant dams would confirm the role of maternal immune activation in structural brain changes throughout development.

MRI has emerged as a versatile tool for studying brain structure in animal models. More recently, the application of this noninvasive technique has been employed to quantitatively study structural changes in the brain, especially in disease states. Volumetric analysis of manually, semi-automatically, or automatically delineated neuroanatomical regions of interest is one of the most commonly used quantitative methods for comparisons between image sets. Whole brain and voxel-based approaches are also popular but are unable to directly examine and compare functionally characterized regions. Longitudinal imaging studies that wish to distinguish normative neurodevelopment from aberrant neurodevelopment typically benefit from using an ROI-based approach. Unfortunately, ROI-based manual segmentation is difficult and time-consuming. Moreover, the ROIs analyzed are often limited to a few brain regions which have been determined based on segmentation practicality or previous literature. To overcome this limitation, we first employed automatic segmentation to simultaneously delineate 26 different ROIs and extract segmented boundaries for volumetric analysis. This method served as an explorative survey into which brain regions were impacted by ZIKV neuropathology throughout development. Our preliminary data from the automated segmentation suggested subtle neuroanatomical effects of prenatal ZIKV infection which we sought to complement and extend upon through manual segmentation.

Currently, magnetic resonance (MR) atlases are unable to entirely recapitulate traditional histological atlases, which can primarily be attributed to insufficient resolution and contrast. These constraints limit the range of structures that can be easily and accurately identified in MR images of the rat brain. Therefore, we also exploited manual segmentation to further delineate subregions such as the dentate gyrus, CA1, CA2, and CA3 within the hippocampus. We were unable to further segment the isocortex as its subregions are not easily identifiable during early postnatal development. In addition to further segmenting prominent brain regions with identifiable subregions, our dual approach involving manual segmentation was necessary to evaluate the accuracy of our proposed automated segmentation scheme. Manual segmentation is considered the gold standard and more precise than automated segmentation. More importantly, it is frequently used to demonstrate the validity of automated methods of segmentation so that more efficient segmentation can be employed for large-scale studies where manual methods are inefficient or not feasible.

Significant challenges remain in the application of the MRI technique to quantitatively study changes in postnatal rodent brain development. Furthermore, preclinical automated segmentation is especially challenging due to the limited number of existing segmentation strategies available to researchers [48]. In addition, the lack of quantitative neurodevelopmental MR atlases challenges researchers who aim to employ automated segmentation for neonatal and juvenile rats. To the best of our knowledge, our study is the first to apply an atlas-based automated segmentation method to both neonatal and juvenile rats. As a consequence, there were limitations to this study. Our proposed automated segmentation scheme utilizing EVA software with the magnetic resonance histology (MRH) atlas of postnatal rat brain development from the Duke Center for In Vivo Microscopy had drawbacks when applied to our previously established paradigm. Most notable was the age discrepancy of the MRH atlas used for the automated segmentation of the P16 brains and the P60 brains. We were unable to find any age-matched MR atlases corresponding with these age points; hence, the P18 and P80 MRH atlases were used respectively for our P16 and P60 MR images. Furthermore, special consideration must be applied to the P16 data due to the rapid brain development that occurs during the period from P2 to P25 [49]. In contrast, brain mass in adulthood is not significantly different from the mass at P60 [49], suggesting that the P80 MR atlas is somewhat comparable to the P60 MR images. Moreover, the experimental data presented in this paper are derived from a different rat subspecies than the MRH atlas [50]. This limits the validity of the automated segmentation, as previous literature has illustrated significant anatomic differences [51,52,53] and differing growth rates [54,55] between laboratory rat strains. Furthermore, the regional volume measurements of the MRH atlas are compiled from five male rats with high morphologic variability in the midbrain [50]. There also has been growing evidence that suggests sexual dimorphism in relation to overall brain and individual structure size within rats [56]. Taken together, the lack of female representation within the atlas is problematic for studies that wish to explore sex differences within their model. Overall, atlas-based segmentation depends largely on the compatibility of the reference volumes and experimental design. Consequently, these observed limitations are attributable to our reliance on the only existing neurodevelopmental MR atlas for rats. 

## 5. Conclusions

In summary, prenatal ZIKV infection alters growth in brain regions throughout the neonatal and juvenile developmental periods. In particular, we demonstrate that maternal ZIKV inoculation is associated with transient increases and decreases in hippocampal subregions during the neonatal and juvenile age points, suggesting altered growth trajectory in these regions during development. Our findings also suggest that maternal immune activation may play a role in altered brain growth. While volume is not a direct measure of brain function, it can be useful in identifying abnormal neurodevelopment and thus be utilized as a potential biomarker for neurological sequelae. Furthermore, the novel methodological approach described in this paper lays the groundwork for future MRI-based studies of postnatal neurodevelopment assessing regional volume differences between normal and disease states. We hope that future studies will improve upon existing strategies for rodent segmentation throughout neurodevelopment so that MR imaging can be further integrated into other rat models of early-life infections. 

## Data Availability

The data presented in this study are available upon request.

## Supplementary Materials

The following are available online at https://www.mdpi.com/article/10.3390/v13061123/s1, Table S1: Average volumes (mm^3^) for the whole brain compared across groups for both methods of segmentation, Table S2: Average corrected volumes (mm^3^) for each individual ROI compared across groups for both methods of segmentation, Table S3: Average corrected volumes (mm^3^) for each hippocampal subregion compared across groups, Table S4: Comparative analysis of ROI volumes (mm^3^) determined by automated and manual segmentation methods for each age point, Figure S1: Bland–Altman plots comparing automated segmentation to manual segmentation.
