# Supplementary material for: An Examination of the Long-Term Neurodevelopmental Impact of Prenatal Zika Virus Infection in a Rat Model Using a High Resolution, Longitudinal MRI Approach
Source: Viruses. 2021 Jun 11;13(6):1123. doi: 10.3390/v13061123 (PMC8230645; doi:10.3390/v13061123)
Supplement: Supplementary file 1 [file viruses-13-01123-s001.zip › viruses-1159135-supplementary.pdf]

Supplementary material

Table S1. Average volumes (mm^3) for the whole brain compared across groups for both methods of segmentation. The average volume ± SD for each brain region at each age is listed.

Segmentation Method	Time point	Mean ±SD		
		Mock	iZIKV	ZIKV
Automated	PND2	354.04 ±43.27	304.97 ±40.28[*,b]	350.57 ±23.03
	PND16	1343.14 ±53.47	1366.13 ±44.77	1382.33 ±58.46*
	PND24	1579.23 ±74.96	1569.66 ±72.68	1581.49 ±85.97
	PND60	1991.82 ±119.01	2008.24 ±127.66	1972.08 ±103.77
Manual	PND2	493.81 ±54.21	370.70 ±33.23[*,b]	427.57 ±31.37*
	PND16	1502.35 ±45.42	1465.72 ±56.52*	1458.32 ±51.49*
	PND24	1677.60 ±42.99	1671.22 ±71.05	1632.60 ±64.27*
	PND60	2128.35 ±124.92	2147.50 ±136.99	2078.42 ±139.26

Significant differences compared with mock group*, iZIKV[a], or ZIKV[b] ($p < 0.05$ by two-way ANOVA and Tukey post-hoc test).

Table S2. Average corrected volumes (mm^3) for each individual ROI compared across groups for both methods of segmentation. The average volume ± SD for each brain region at each age is listed.

Brain Region	Segmentation Method	Time point	Mean ±SD		
			Mock	iZIKV	ZIKV
Cerebellum	Automated	P2	3.53 ±0.37	3.36 ±0.34	3.33 ±0.30
		P16	13.06 ±0.75	12.35 ±0.63*	12.30 ±0.50*
		P24	13.48 ±0.49	13.24 ±0.96	13.22 ±0.58
		P60	15.39 ±0.30	15.18 ±0.45	15.24 ±0.47
	Manual	P2	8.03 ±0.78	7.33 ±0.77*	7.91 ±0.92
		P16	14.15 ±0.70	14.05 ±0.43	13.89 ±0.53
		P24	14.75 ±0.47	15.10 ±0.52	14.60 ±0.63[a]
		P60	14.55 ±0.57	15.15 ±0.65	14.71±0.65
Isocortex	Automated	P2	24.89 ±1.87	24.30 ±1.07	23.74 ±1.25*
		P16	33.47 ±1.29	32.40 ±1.05*	32.03 ±1.06*
		P24	33.31 ±1.11	32.95 ±1.35	32.53 ±1.28
		P60	28.83 ±0.56	28.83 ±0.68	28.40 ±0.86
	Manual	P2	33.27 ±1.85	30.23 ±1.46[*,b]	33.39 ±1.78
		P16	34.92 ±0.82	33.72 ±0.85[*,b]	34.61 ±0.82
		P24	34.57 ±0.75	34.00 ±0.62	34.16 ±0.86
		P60	30.55 ±0.56	30.21 ±0.71	30.24 ±0.86
Hippocampus	Automated	P2	4.99 ±0.56	5.01 ±0.24	5.02 ±0.55
		P16	5.42 ±0.14	5.43 ±0.19	5.25 ±0.21[*,a]
		P24	5.42 ±0.21	5.41 ±0.27	5.23 ±0.28
		P60	5.36 ±0.13	5.39 ±0.12	5.24 ±0.22[a]
	Manual	P2	2.35 ±0.42	2.16 ±0.23	2.073 ±0.33
		P16	2.85 ±0.30	2.87 ±0.26	3.18 ±0.46[*,a]
		P24	4.23 ±0.45	4.36 ±0.45	4.45 ±0.51
		P60	4.23 ±0.31	4.33 ±0.45	4.32 ±0.38

Significant differences compared with mock group*, iZIKV[a], or ZIKV[b] ($p < 0.05$ by two-way ANOVA and Tukey post-hoc test).

Table S3. Average corrected volumes (mm³) for each hippocampal subregion compared across groups. The average volume ± SD for each brain region at each age is listed.

Subregion	Age Point	Mean ±SD		
		Mock	iZIKV	ZIKV
CA1	P2	0.7145 ±0.1228	0.6520 ±0.0934	0.6963 ±0.1442
	P16	0.9096 ±0.2288	0.9306 ±0.2231	1.1544 ±0.3811*
	P24	1.4015 ±0.2511	1.6014 ±0.3027	1.6253 ±0.3604
	P60	1.3787 ±0.1447	1.4596 ±0.1638	1.4655 ±0.2357
CA2	P2	0.2166 ±0.1758	0.1550 ±0.0422	0.1464 ±0.0324
	P16	0.1857 ±0.0655	0.1747 ±0.0724	0.1728 ±0.0609
	P24	0.2262 ±0.0509	0.1948 ±0.0540	0.1919 ±0.0467
	P60	0.2158 ±0.0391	0.2131 ±0.0578	0.1810 ±0.0672
CA3	P2	0.6264 ±0.1160	0.5873 ±0.1290	0.5228 ±0.0682*
	P16	0.6653 ±0.1009	0.6672 ±0.0847	0.6778 ±0.1250
	P24	0.8763 ±0.1938	0.9176 ±0.2554	0.9820 ±0.2449
	P60	0.8549 ±0.1383	0.9565 ±0.1574	0.9366 ±0.1571
DG	P2	0.6327 ±0.1095	0.5884 ±0.0609	0.5572 ±0.0996
	P16	0.7115 ±0.1362	0.7688 ±0.1337	0.8378 ±0.1694*
	P24	1.1418 ±0.1238	1.1410 ±0.1492	1.1837 ±0.1113
	P60	1.2438 ±0.0818	1.1548 ±0.0993	1.2188 ±0.1354

Significant differences compared with mock group*, iZIKV[a], or ZIKV[b] ($p < 0.05$ by two-way ANOVA and Tukey post-hoc test).

Table S4. Comparative analysis of ROI volumes (mm³) determined by automated and manual segmentation methods for each age point. The average uncorrected volume ± SD for each segmentation method is listed for CB, CTX, HC, and WB regions.

Age Point	ROI	Mean ±SD		Pearson *r*	*p*-value
		Automated	Manual		
P2	Cerebellum	11.47 ±1.62	33.79 ±6.96	0.659***	5.91E-08
	Cortex	81.97 ±10.66	140.97 ±28.05	0.794***	7.85E-13
	Hippocampus	16.80 ±1.99	9.59 ±2.54	0.377*	1.28E-02
	Whole Brain	337.44 ±42.31	432.97 ±65.02	0.684***	1.16E-08
P16	Cerebellum	171.34 ±7.77	207.24 ±12.86	0.429***	8.61E-04
	Cortex	81.97 ±10.66	508.40 ±24.17	0.624***	2.14E-07
	Hippocampus	444.71 ±13.78	43.83 ±5.78	-0.027	8.44E-01
	Whole Brain	1363.46 ±54.29	1476.11 ±53.94	0.223	9.51E-02
P24	Cerebellum	209.51 ±8.96	245.45 ±15.36	0.109	4.38E-01
	Cortex	519.87 ±27.24	567.61 ±26.03	0.346*	1.10E-02
	Hippocampus	84.35 ±5.00	72.24 ±7.97	0.124	3.77E-01
	Whole Brain	1576.83 ±77.25	1658.45 ±63.85	0.099	4.80E-01
P60	Cerebellum	303.77 ±17.19	312.83 ±22.94	0.363**	8.40E-03
	Cortex	570.35 ±34.34	641.71 ±42.10	0.587***	5.00E-06
	Hippocampus	105.93 ±7.04	90.64 ±7.60	0.383**	5.00E-03
	Whole Brain	1989.66 ±115.16	2116.00 ±134.72	0.465**	1.00E-03

Pearson correlation coefficients (r) are reported with p-value (*$p < 0.05$, **$p < 0.01$, ***$p < 0.001$).

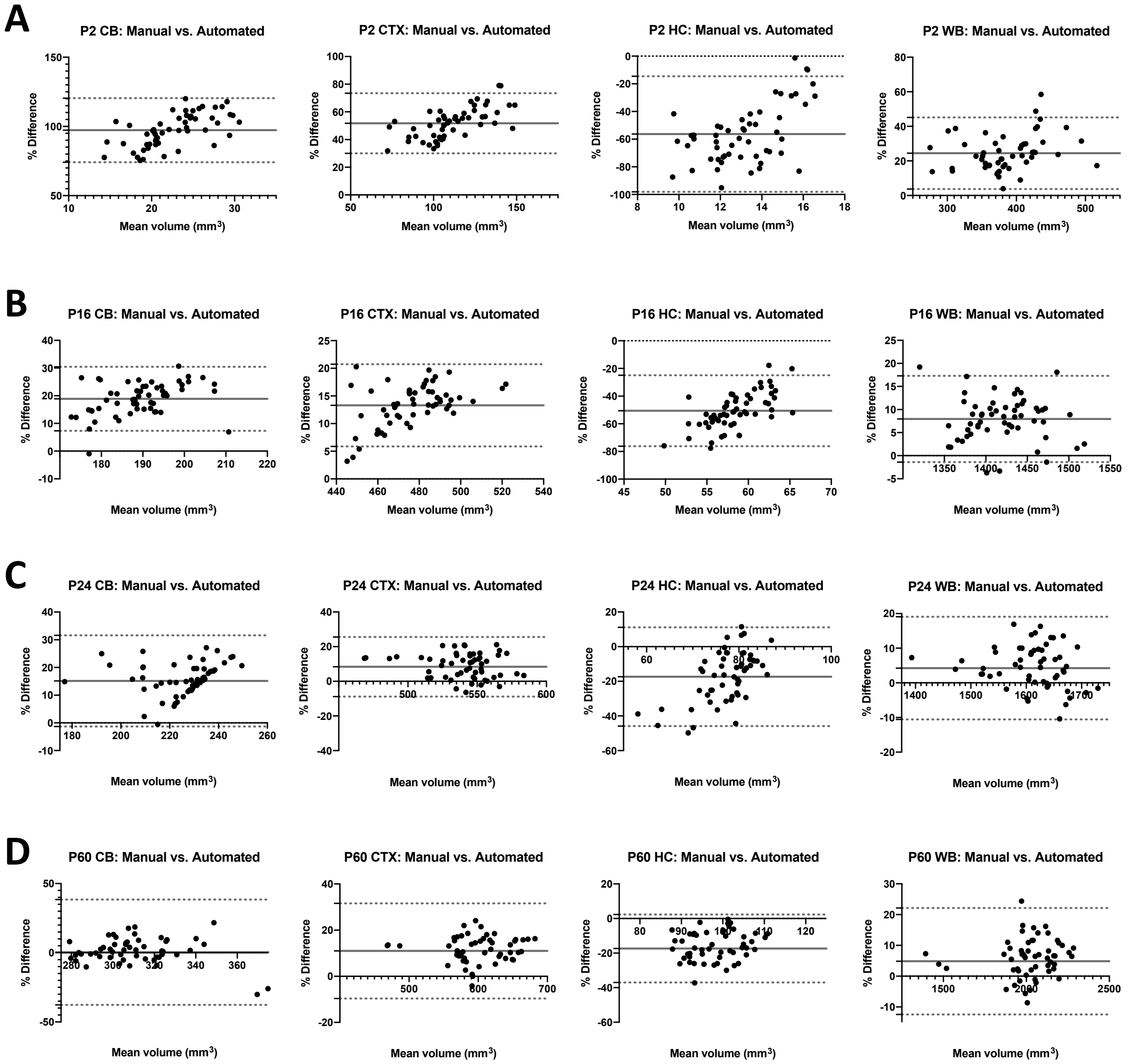

Figure S1. Bland-Altman plots comparing automated segmentation to manual segmentation. Percent volumetric difference between measurements using automated and manual segmentation was plotted against the average. Each brain structure, including the whole brain, was compared for postnatal day 2 (**A**), postnatal day 16 (**B**), postnatal day 24 (**C**), and postnatal day 60 (**D**). Solid line represents the mean and the dashed lined represent the 95% limits of agreement (±1.96SD, indicated by the area between the dashed lines).
